# Phase 1 clinical trial of the ataxia telangiectasia and Rad3-related inhibitor berzosertib with irinotecan in patients with advanced solid tumors (ETCTN 9938)

**DOI:** 10.1002/cncr.70157

**Published:** 2025-11-01

**Authors:** Liza C. Villaruz, Jingxiao Jin, Hong Wang, James J. Lee, Jason J. Luke, John C. Rhee, Saiama N. Waqar, Geoffrey I. Shapiro, James Cleary, Diwakar Davar, Timothy F. Burns, Rashmi Verma, Manik Amin, Leonard Appleman, Kristen K. Ciombor, Elizabeth J. Davis, Jonathan W. Riess, E. Claire Dees, Edward J. Kim, Dana Cardin, Jianxia Guo, Susan M. Christner, Joshua Deppas, Angelina M. Parise, John C. Schmitz, Bella S. Guerrouahen, Michael Suchko, Alejandro Contreras, Li Li, Katherine V. Ferry-Galow, Ting-Chia Chang, Shahanawaz Jiwani, Christopher J. Bakkenist, Edward Chu, Steven D. Gore, Jan H. Beumer

**Affiliations:** 1 Cancer Therapeutics Program, UPMC Hillman Cancer Center, Pittsburgh, Pennsylvania, USA; 2 Division of Hematology/Oncology, Department of Medicine, School of Medicine, University of Pittsburgh, Pittsburgh, Pennsylvania, USA; 3 Department of Biostatistics, School of Public Health, University of Pittsburgh, Pittsburgh, Pennsylvania, USA; 4 Washington University School of Medicine, St. Louis, Missouri, USA; 5 Dana-Farber Cancer Institute, Boston, Massachusetts, USA; 6 UC Davis Comprehensive Cancer Center, Sacramento, California, USA; 7 University of Chicago Comprehensive Cancer Center, Chicago, Illinois, USA; 8 Vanderbilt University Medical Center, Nashville, Tennessee, USA; 9 Division of Oncology, Department of Medicine, University of North Carolina, Chapel Hill, North Carolina, USA; 10 Program in Oncology, University of Maryland Marlene and Stewart Greenebaum Comprehensive Cancer Center, Baltimore, Maryland, USA; 11 Division of Pathology and Laboratory Medicine, The University of Texas MD Anderson Cancer Center, Houston, Texas, USA; 12 Frederick National Laboratory for Cancer Research, Clinical Pharmacodynamics Biomarker Program, Applied/Developmental Research Directorate, Frederick, Maryland, USA; 13 Molecular Characterization Laboratory, Frederick National Laboratory for Cancer Research, Frederick, Maryland, USA; 14 Department of Radiation Oncology, UPMC Hillman Cancer Center, School of Medicine, University of Pittsburgh, Pittsburgh, Pennsylvania, USA; 15 Montefiore Einstein Cancer Canter, Bronx, New York, USA; 16 Division of Cancer Treatment and Diagnosis, Investigational Drug Branch, Cancer Therapy Evaluation Program, National Cancer Institute, Bethesda, Maryland, USA; 17 The Sidney Kimmel Cancer Center at Johns Hopkins, Baltimore, Maryland, USA

**Keywords:** ATR inhibition, berzosertib, irinotecan, phase 1, solid tumors

## Abstract

**Background:**

Ataxia telangiectasia and Rad3-related (ATR) inhibition with berzosertib potentiates the efficacy of irinotecan in preclinical models. The authors hypothesize that this combination is well tolerated, modulates the DNA damage response to irinotecan, and is associated with clinical activity in advanced solid tumors.

**Methods:**

In this phase 1 study (NCT02595931), berzosertib 60–270 mg/m^2^ was administered with irinotecan 180 mg/m^2^ every 2 weeks in a 4-week cycle. The primary end point was determination of the maximum tolerated dose and recommended phase 2 dose (RP2D). Antitumor activity, pharmacokinetics, and pharmacodynamics were secondary end points.

**Results:**

Sixty-three patients were enrolled, the majority with colorectal cancer (49%) or pancreatic cancer (21%). Median number of prior lines of therapy was four (range, 2–7). Two dose-limiting toxicities, both febrile neutropenia, occurred among 45 patients treated with berzosertib 270 mg/m^2^ and irinotecan 180 mg/m^2^. The most common treatment-related grade ≥3 toxicities were lymphopenia (30%), neutropenia (29%), and anemia (25%). Two partial responses occurred in patients with pancreatic cancer and ataxia telangiectasia mutated (ATM) alterations: 32% decrease in an ATM E11828/ATM K1109* tumor lasting 15.8 months and 57% decrease in an ATM R3008H/germline ATM R1882* tumor lasting 13.6 months. Induction of DNA damage markers γH2AX and pNBS1 was observed in one tumor biopsy obtained post berzosertib and irinotecan combination treatment compared with post irinotecan alone.

**Conclusion:**

Berzosertib 270 mg/m^2^ and irinotecan 180 mg/m^2^ was the RP2D. The combination is associated with manageable side effects and promising disease activity in ATM mutant solid tumors.

## INTRODUCTION

The DNA damage response (DDR) is a network of signaling pathways that integrate genomic stability and cell viability and is an attractive target for sensitizing cancer cells to chemotherapy.^1,2^ The primary regulators of the DDR network are ataxia telangiectasia Rad3-related (ATR) and ataxia telangiectasia mutated (ATM) protein kinases. ATR acts predominantly in the S/G2 phases of the cell cycle and is recruited to areas of single-stranded DNA, which occur at damaged replication forks and lesions undergoing homologous recombination repair. ATR phosphorylates and activates checkpoint kinase 1 (CHK1) and ATR and CHK1 kinase signaling mediate transient cell-cycle arrest and DNA repair. In contrast, ATM is recruited to DNA double strand breaks (DSB) and phosphorylates and activates checkpoint kinase 2 (CHK2), and ATM and CHK2 kinase signaling mediate the G1/S checkpoint through downstream phosphorylation of p53 and DSB repair.^3^

Unlike normal cells, cancer cells often exhibit defects in ATM-p53 signaling.^3^ Loss of the G1 checkpoint may render tumor cells more dependent on ATR-mediated cell-cycle checkpoints and repair in S- and G2-phases of the cell cycle for cell survival.^2–4^ Thus, ATR inhibition may preferentially kill malignant cells with defects in ATM and p53 while sparing normal tissue.

Topotecan and irinotecan are camptothecin-derived topoisomerase I (Top1) inhibitors with activity in a variety of solid tumor malignancies. Top1 is an essential enzyme that unwinds supercoiled DNA during replication through formation of transient single strand DNA breaks.^5^ Top1 inhibitors trap Top1 cleavage complexes, generating potentially lethal replication-associated DSBs when a DNA polymerase collides with a single strand break in a stable complex on the opposing strand. Consistent with its role in the repair of damaged replication forks and homologous recombination repair, depletion of ATR was found to sensitize cells to Top1 inhibitors in a genome wide genetic screen.^6^ Furthermore, ATR inhibition potentiated the antitumor effect of irinotecan in preclinical models of colorectal cancer (CRC) leading to substantial tumor regression.^6^ Berzosertib (M6620, previously VX-970) is a highly potent and selective ATP-competitive inhibitor of ATR.^1^ A phase 1 trial assessing berzosertib monotherapy demonstrated that it was well tolerated with no dose-limiting toxicities (DLTs) at the highest dose level (DL) of 240 mg/m^2^ once or twice weekly.^7^

We conducted a phase 1 clinical trial of berzosertib combined with irinotecan in patients with advanced solid tumors with the primary objective of determining the maximum tolerated dose (MTD) and recommended phase 2 dose (RP2D) of this novel drug combination. Secondary objectives were to estimate the safety and tolerability of berzosertib in combination with irinotecan, as well as the overall response rate (ORR) and progression-free survival (PFS). In addition, we characterized the pharmacokinetic (PK) and pharmacodynamic parameters of berzosertib in combination with irinotecan.

## MATERIALS AND METHODS

### Patients

This trial was conducted under a National Cancer Institute (NCI) Center for Cancer Research–sponsored investigational new drug application with institutional review board approval (ClinicalTrials.gov ID: NCT02595931). Written informed consent was obtained from enrolled patients.

Eligible patients had histologically confirmed metastatic or unresectable malignancy that was refractory to standard therapy or for which no standard therapy was available and for whom irinotecan was deemed a reasonable treatment option. Patients had measurable disease based on RECIST v1.1, Eastern Cooperative Oncology Group (ECOG) performance status of 0 or 1, and adequate organ and marrow function.

### Study design and treatment

Patients with advanced malignancies were administered berzosertib plus irinotecan intravenously (iv) in this open-label, single-arm, phase 1 study. Berzosertib was supplied under a Collaborative Research and Development Agreement among NCI, Vertex Pharmaceuticals, and EMD Serono. Irinotecan was obtained from commercial sources.

Treatment cycles were 28 days long (Supporting Information S1: Figure S1). Irinotecan alone was administered as a PK lead-in on day −14 before cycle 1 (C1) only. From C1 onward, irinotecan was administered at the assigned DL (150 to 180 mg/m^2^) iv on days 1 and 15. Berzosertib was administered iv within 30 minutes after completion of irinotecan at the assigned DL (60 to 270 mg/m^2^) on days 1 and 15.

After determination of MTD, an expansion cohort of 15 solid tumor patients was enrolled at the RP2D. The expansion cohort was limited to patients with known deficiencies in the DDR, e.g., mutations in ATM, PALB2, BRCA1/2, or patients with the following tumor types regardless of known DDR deficiency: pancreatic cancer, CRC, and small cell lung cancer. Patients in the expansion cohort underwent mandatory paired tumor biopsies obtained at cycle 1 day −13 (C1D-13) and cycle 1 day 2 (C1D2) for pharmacodynamics assessment.

### Safety and efficacy evaluations

A history and physical examination were conducted at baseline and before each cycle. Complete blood counts and serum chemistries were performed at baseline and weekly during C1 and every 2 weeks thereafter. Toxicities were graded using NCI Common Terminology Criteria for Adverse Events (version 5.0) and defined as adverse events that were possibly, probably, or definitely related to treatment. Radiographic evaluation was performed at baseline and every 8 weeks for tumor response based on Response Evaluation Criteria in Solid Tumors (RECIST) version 1.1.

### Statistics

DLs were evaluated using a modified Storer’s up and down design with the plan of enrolling up to 36 evaluable patients during dose escalation (Supporting Information S1: Figure S1).^8^ Stage I of the Modified Storer’s up and down design is single patient dose escalation until a DLT is observed. Once a DLT is observed, patients are treated in cohorts of five in stage II of the design. The MTD was defined as the highest DL tested at which ≤20% patients experience dose limiting toxicity (DLT) during C1 of therapy.

Toxicities and responses were tabulated. PFS was estimated using the Kaplan–Meier method. Statistical analyses were performed using SAS (version 9.4, SAS Institute Inc, Cary, North Carolina).

### Pharmacokinetics

Because the irinotecan active metabolite SN-38 is metabolized by UGT1A1, a baseline blood sample was obtained for UGT1A1*28 genotyping. Patients were sampled for EDTA plasma PK of berzosertib on cycle 1 day 15 (C1D15), and PK of irinotecan and metabolites SN38, SN38G, APC, and NPC, on day −14 before C1 and C1D15. Plasma was collected before and at 30, 60, and 85 minutes after the start, and 5, 15, 30, and 55 minutes after the end of the 90-minute irinotecan infusion (approximately 55 min after start of the 1-hour berzosertib infusion), and 5, 15, and 30 minutes, and 1, 2, 4, 21.5, 45.5, and 69.5 hours after the end of the berzosertib infusion.

Each blood sample was centrifuged at approximately 1000*g*, and plasma was stored at −70°C or colder until analysis for berzosertib with a liquid chromatography-tandem mass spectrometry (LC-MS/MS) assay validated to Food and Drug Administration guidance,^9^ and for irinotecan and metabolites with an LC-MS/MS assay based on a prior published assay.^10^ Modifications included the use of stable isotope versions: [D_10_]-Irinotecan, [D_3_]-APC, [D_3_]-SN-38 (Toronto Research Chemicals, Toronto, Canada), and [D_6_]-SN38G (ALSACHIM, Paris, France). Berzosertib plasma protein binding was assessed in vitro using rapid equilibrium dialysis (RED) devices (Thermo Fisher Scientific, Waltham, Massachusetts).

Plasma pharmacokinetic parameters were derived from the data by noncompartmental methods with WinNonlin (Certara). Statistical analyses for pharmacokinetic parameter values were performed using SPSS 27.0 for Windows (SPSS Inc, Chicago, Illinois).

### Pharmacodynamics

#### Peripheral blood mononuclear cell DNA damage signaling

Quasi-quantitative dual multiplexed immunoblot analysis was performed to determine induction of DNA damage in peripheral blood mononuclear cells (PBMCs) at baseline, 85 min, 4.5 hours, and 24 hours after the start of the irinotecan infusion on cycle 1 day −14 (C1D-14) and C1D15. Blood was processed and analysis performed as described.^11–13^

#### Tumor DNA damage signaling

Paired tumor biopsies were collected from patients enrolled in the expansion cohort. Biopsies were obtained at 18–22 hours after the end of irinotecan infusion (C1, day −13) and 18–22 hours after the end of irinotecan and berzosertib combination treatment (C1, day 2), then analyzed for levels of biomarkers γH2AX, pS343-NBS1, and pS824-KAP1 using the quantitative multiplex immunofluorescence assays validated for use on formalin-fixed, paraffin-embedded (FFPE) human tissue.^14^

The staining of FFPE tissue sections used the BOND RX autostainer (Leica Biosystems). For γH2AX and pS343-NBS1 analysis, biotin-conjugated γH2AX (clone JBW301; EMD Millipore) and a custom-conjugated pS343-NBS1-DIG (clone EP178; Abcam) antibodies were detected with the use of streptavidin labeled with Alexa Fluor 488 and Alexa Fluor 647 custom-conjugated IgG Fraction Monoclonal Mouse Anti-Digoxin antibody, respectively. For pS824-KAP1 analysis, anti-pS824-KAP1 antibody (clone EPR5248; Abcam) custom conjugated to dinitrophenol (Molecular Probes, Inc) was detected using Alexa Fluor 488 conjugated anti-dinitrophenyl KLH rabbit polyclonal antibody. In addition, β-catenin antibody custom conjugated to Alexa Fluor 546 was used to define tumor areas in all biomarker analyses. For γH2AX and pS343-NBS1 analysis, fluorescent whole slide images were acquired at 20× using Leica’s Aperio FL scanner. Images were then analyzed using a custom algorithm build in Definiens Tissue Studio Analysis software (formerly Definiens AG). For pS824-KAP1 analysis, whole slide fluorescent images were acquired at 20× using ZEISS Axioscan 7 scanner. Images were analyzed using a custom algorithm in HALO software (Indica Labs).

### Whole exome sequencing

Archival tumor specimens were collected for whole exome sequencing (WES) as part of an exploratory analysis to identify molecularly defined populations particularly responsive to berzosertib and irinotecan.

WES and bioinformatics were performed as previously described.^15,16^ Aneuploidy score was derived from WES data and defined as the number of chromosome arms with aneuploidy (range: 0–39), excluding acrocentric arms. Arm-level aneuploidy was identified if more than 90% of an arm had gained or lost copy number, defined by an absolute fold change in segment mean greater than 0.3.^17^

## RESULTS

### Patient characteristics

Between July 2016 and July 2021, 63 patients were enrolled, of whom 61 received study drug treatment (Table 1). The median number of prior lines of therapy was four (range, 2–7). Patients most commonly had CRC (49%) or pancreatic cancer (21%), non–small cell lung cancer (5%), and small cell lung cancer (5%). Twenty-one patients (33%) had UGT1A1 *1/*28 genotype and six patients (10%) had *28/*28 genotype. Forty-nine patients completed at least one cycle (median, 2 cycles; range, 1–9 cycles) of treatment and were evaluable for DLT.

### Dose escalation and determination of MTD and recommended phase 2 dose

In stage I, one patient experienced DLT at dose level 3 with a grade 3 lung infection. In stage II, four of the first 11 patients treated at dose level 4 (DL4) were unable to complete the DLT evaluation period due to clinically significant toxicity not meeting DLT criteria; one patient each experienced grade 2 diarrhea and grade 3 diarrhea, and two patients experienced grade 3 neutropenia. The protocol was thereafter amended to limit dose escalation to DL4, and the remaining patients for the dose escalation portion were enrolled at DL4. An additional 15 patients were enrolled at DL4 as part of the dose expansion. At DL4, a total of 45 patients were enrolled, 33 of whom completed one cycle of therapy and were evaluable for DLT. In addition to the four patients unable to complete (C1) of therapy due to clinical significant toxicity not meeting DLT criteria described above, four patients experienced clinical progression of their disease, one patient developed neutropenic fever after the day −14 irinotecan lead-in, one patient experienced hypersensitivity to irinotecan, and two patients withdrew consent after the irinotecan lead-in; thus a total of 12 patients were unevaluable for DLT at DL4. At DL4, there were two DLTs, both of which were febrile neutropenia. Berzosertib 270 mg/m^2^ and irinotecan 180 mg/m^2^ every 2 weeks (DL4) were established as the MTD and RP2D.

### Toxicity

The most common treatment-related toxicities across all DLs (*N* = 63) were anemia (57%), lymphopenia (54%), leukopenia (52%), diarrhea (52%), neutropenia (49%), nausea (41%), vomiting (37%), fatigue (32%), and thrombocytopenia (25%) (Table 2). The most common treatment-related grade ≥3 toxicities were lymphopenia (30%), neutropenia (29%), anemia (25%), leukopenia (22%), and diarrhea (16%).

Twelve patients required one dose reduction for grade 3 neutropenia (five patients), grade 3 febrile neutropenia (one patient), grade 2 neutropenia (one patient), grade 2 anemia (one patient), grade 3 diarrhea (one patient), grade 2 diarrhea (one patient), grade 2 nausea (one patient), and grade 1 nausea (one patient). Four patients required a second dose reduction for grade 4 neutropenia (one patient), grade 2 thrombocytopenia (one patient), grade 2 diarrhea (two patients), and grade 2 fatigue (one patient).

Four patients discontinued treatment due to adverse events: grade 2 diarrhea (one patient), grade 3 febrile neutropenia (one patient), grade 4 febrile neutropenia (one patient), and grade 1 diarrhea, nausea, and creatinine increase (one patient).

### Efficacy

**FIGURE 1:**
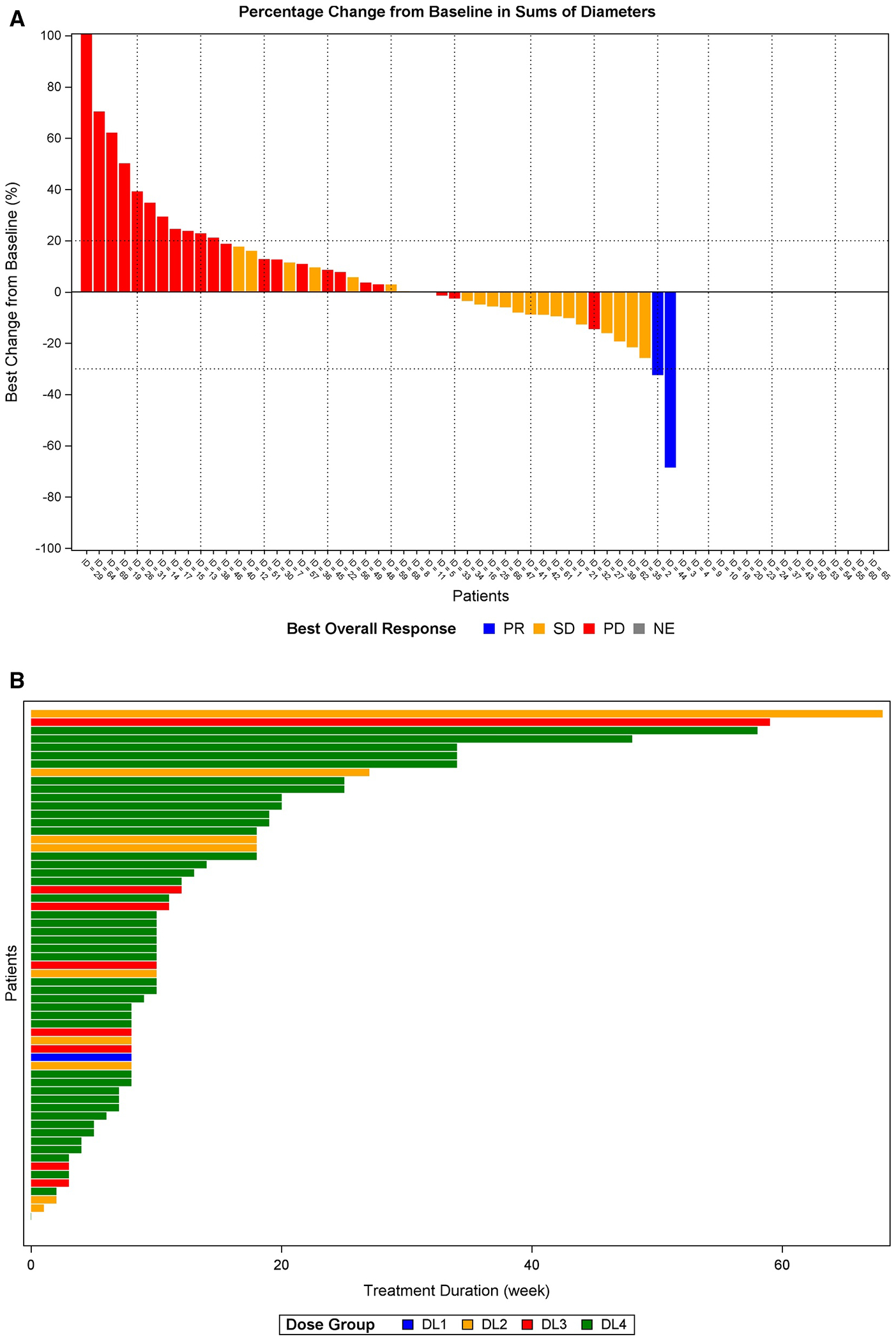
Efficacy in the overall population. (A) The waterfall plot shows responses in evaluable patients (*n* = 49). (B) The swimmer plot shows duration of treatment in evaluable patients. Computed tomography scans at baseline and on treatment show a partial response in (C) a patient with an ATM E11828/ATM K1109* pancreatic adenocarcinoma and (D) a patient with an ATM R3008H/germline ATM R1882* pancreatic adenocarcinoma.

Of 49 patients evaluable for response, two had a confirmed partial responses (PR), 22 had stable disease (SD), and 25 had progressive disease (PD) as best response (Figure 1A). The ORR was 4% (95% confidence interval [CI], 1%–14%) and median PFS was 3 months (95% CI, 2–4). Notably, the two confirmed PRs both occurred in patients with metastatic pancreatic cancer whose tumors harbored pathogenic ATM alterations, found on local next generation sequencing (NGS) testing. The first patient was a 60-year-old female with an ATM E11828/ATM K1109* mutated tumor who had undergone two prior lines of treatment (gemcitabine, nab-paclitaxel, and indoximod, followed by capecitabine and tosedostat). She was treated at DL2 and achieved a 32% decrease in target lesions lasting 15.8 months (Figure 1B,C). The second patient was a 58-year-old female with an ATM R3008H/germline ATM R1882* mutated tumor who had undergone three prior lines of treatment for metastatic disease (gemcitabine, docetaxel, capecitabine, and cisplatin, second-line niraparib and ipilimumab, followed by 5-fluorouracil, leucovorin, and oxaliplatin). She was treated at DL4 and achieved a 57% decrease in target lesions lasting 13.6 months (Figure 1B,D).

### Pharmacokinetics

**FIGURE 2:**
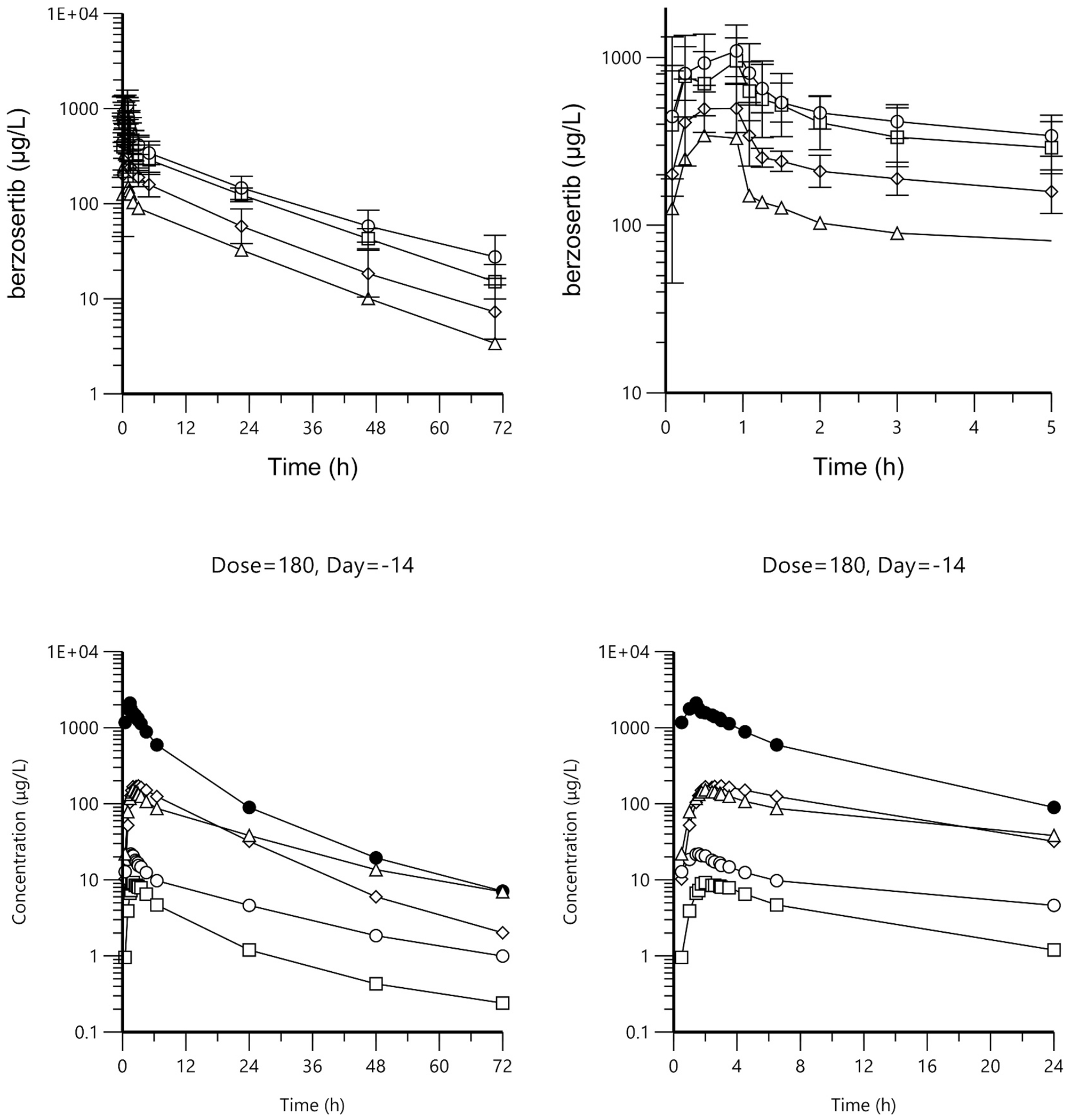
Pharmacokinetic profiles of (A) berzosertib (geometric mean, SD) on day 15 at 60 (△), 120 (◇), 180 (□), and 270 (◯) mg/m^2^ (right panel, 0–5 hour zoom) and (B) irinotecan (•), SN38 (◯), SN38G (△), APC (◇), and NPC (□) (geometric mean) on day −14 at 180 mg/m^2^ (right panel, 0–24 hour zoom).

Berzosertib pharmacokinetic data were available for 39 patients, one at 60 mg/m^2^, five at 120 mg/m^2^, eight at 180 mg/m^2^, and 25 at 270 mg/m^2^ (Supporting Information S1: Table S1; Figure 2A). Berzosertib displayed a biphasic plasma concentration versus time profile, with a half-life of approximately 17 hours, a volume of distribution of approximately 530 L/m^2^ and a total clearance of 24 L/h/m^2^. We observed a trend of increasing half-life with increasing dose (*p* = .005), which could be attributed to an increasing volume of distribution (*p* = .025) at a constant clearance (*p* = .693) (Supporting Information S1: Figure S2). In vitro protein binding suggested an increased free fraction of berzosertib starting at approximately 30 μg/mL (Supporting Information S1: Figure S3).

Irinotecan pharmacokinetic data were available for 55 patients for day −14 all at 180 mg/m^2^, and 41 patients for day 15, 37 at 180 mg/m^2^, and four at 150 mg/m^2^ (Supporting Information S1: Table S2; Figure 2B). Irinotecan displayed a biphasic plasma concentration versus time profile, with a half-life of approximately 13 hours, a volume of distribution of approximately 126 L/m^2^, and a total clearance of 12.9 L/h/m^2^. All metabolites displayed elimination profiles parallel to the irinotecan elimination phase, except SN38G, as expected based on the enterohepatic recycling of this metabolite (Supporting Information S1: Table S3). In our evaluation of the impact of berzosertib on irinotecan pharmacokinetics, we analyzed irinotecan and SN38 area under the curve (AUC) following the bioequivalence approach. The 90% CI of the AUC ratio (presence/absence of berzosertib) for both irinotecan and active metabolite SN38 fell within the traditional boundaries of 80%–125% (Supporting Information S1: Table S2). Next, we calculated the metabolic ratios of SN38/SN38G representing glucuronidation by UGT1A1, of NPC/irinotecan and APC/irinotecan representing CYP3A, and SN38/irinotecan representing carboxylesterase (Supporting Information S1: Table S4). Berzosertib did not appear to impact glucuronidation of SN38 to SN38G, or hydrolysis of irinotecan to SN38. Surprisingly, the impact of berzosertib on CYP3A metabolism as measured by the ratio of APC to irinotecan was not statistically significant, whereas it was statistically significant as measured by the ratio of NPC to irinotecan, with a 21% decreased metabolic ratio (*p* = .005).

We evaluated the impact of UGT1A1 genotype on SN38 and SN38G PK, where only the day −14 SN38G AUC as a function of genotype reached statistical significance (*p* = .026) (Supporting Information S1: Table S5; Figure S4), and on day 15 (during combination treatment), no significance was observed. We did not detect a significant association between irinotecan, SN38, SN38G, or SN38G/SN38 ratio versus toxicity, neither in the lead-in, nor in the combination C1 (Supporting Information S1: Table S6). Interestingly, berzosertib exposure was higher in patients with C1 higher any grade toxicity and higher hematologic grade toxicity (*p* = .035 and *p* = .032) (Supporting Information S1: Table S6; Figure S5). In directly comparing toxicity by UGT1A1 genotype, we found statistical significance in C1 only for any toxicity and nonhematologic toxicity with higher grade toxicity associated with more *28 alleles (Supporting Information S1: Table S7).

### Pharmacodynamics

#### PBMC DNA damage signaling

**FIGURE 3:**
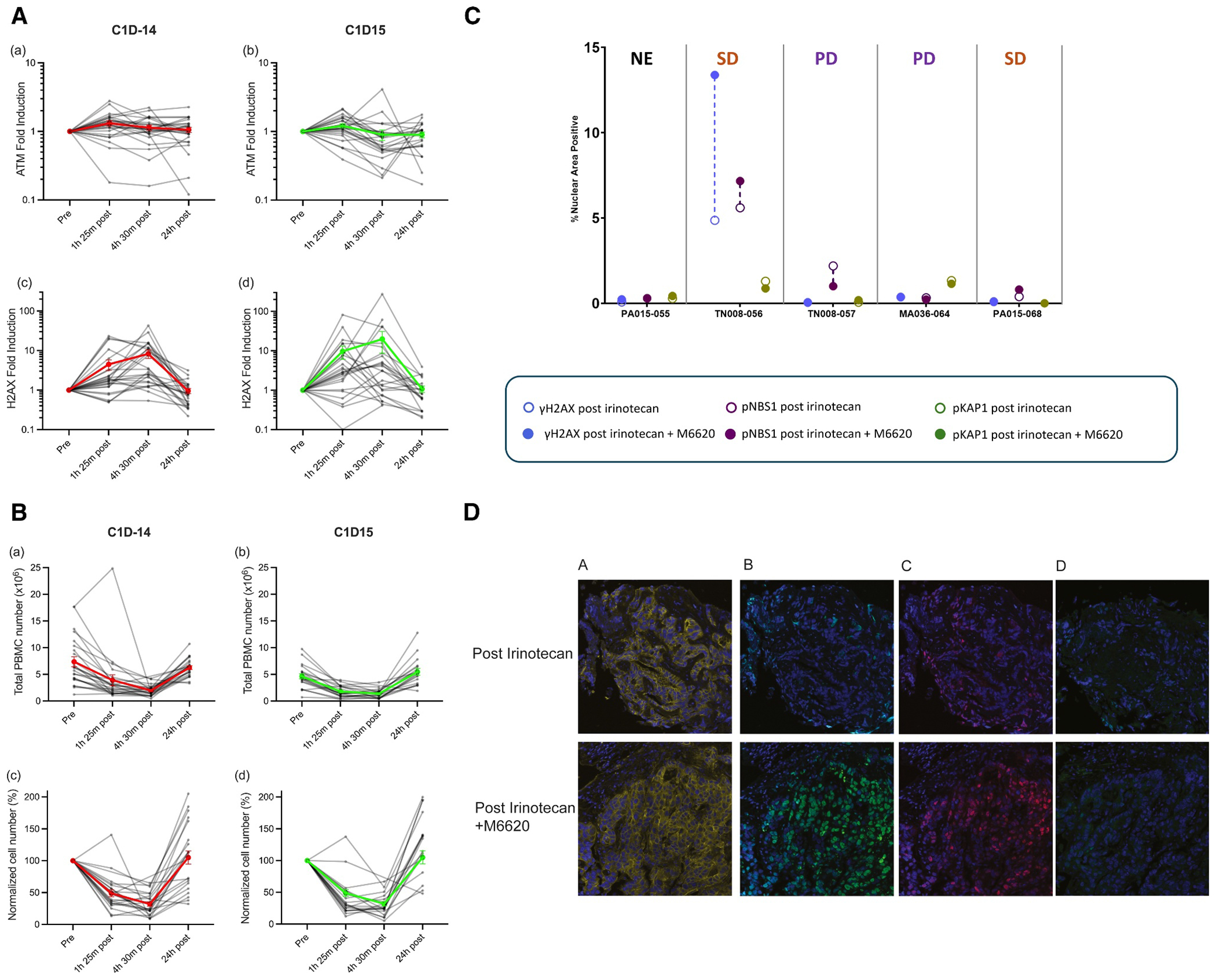
Pharmacodynamic analyses. (A) Induction of ATM and H2AX in PBMCs following (a,c) irinotecan and (b,d) irinotecan in combination with berzosertib. Gray lines represent individual patient results (*n* = 25). Red and green lines represent the mean ±SEM from C1D-14 and C1D15 samples, respectively. (B) PBMC cell numbers and normalized cell numbers (“pre” sample = 100%) following (a,c) irinotecan and (b,d) irinotecan in combination with berzosertib. Gray lines represent individual patient results (*n* = 25). Red and green lines represent the mean ± SEM from C1D-14 and C1D15 samples, respectively. (C) Change in percent NAP values for DDR markers γH2AX, pNBS1 (pS343-NBS1), and pKAP1 (pS824-KAP1) in tumor biopsies collected 18–22 hours post irinotecan single agent (C1D-13) and irinotecan and M6620 combination (C1D2) treatment. Patient responses to the treatment are shown above the graph. (D) Immunofluorescence assays for γH2AX, pNBS1 (pS343-NBS1) and pKAP1 (pS824-KAP1) in paired tumor biopsies from a patient with colon cancer. (a) β-catenin, (b) γH2AX, (c) pNBS1, and (d) pKAP1. β-catenin staining was used to demarcate tumor area. Increases in γH2AX and pNBS1 levels were observed in the post combination treatment biopsy. C1D2, cycle 1 day 2; C1D-13, cycle 1 day −13; C1D-14, cycle 1 day −14; C1D15, cycle 1 day 15; DDR indicates DNA damage response; NAP, nuclear area positive; PBMCs, peripheral blood mononuclear cells.

In patient PBMC samples obtained following administration of irinotecan alone (C1D-14) or in combination with berzosertib (C1D15), no induction of ATM, a marker of DNA DSBs, occurred (Figure 3A). However, we observed a 10-fold induction of H2AX phosphorylation at 4 hours 30 minutes, which returned to baseline levels by 24 hours (Figure 3A). The combination of berzosertib and irinotecan did not increase the DNA damage signal compared to irinotecan alone. Notably, during blood collection, we noticed a striking 70% decrease in PBMC number during and after irinotecan administration (Figure 3B). By 24 hours post administration, the PBMC number returned to baseline.

#### Tumor DNA damage signaling

Paired tumor biopsies were collected from 11 patients enrolled in the expansion cohort, including patients with CRC, pancreatic cancer, small cell lung cancer (SCLC), or DNA damage repair deficient tumors, at 18–22 hours after the end of irinotecan infusion (C1D-13) and 18–22 hours after the end of irinotecan and berzosertib combination treatment (C1D2). Five paired tumor biopsies showed adequate tumor content to be analyzable and reportable for DNA damage response biomarkers (γH2AX, pS343-NBS1, and pS824-KAP1) at both time points (Figure 3C). Among the five sets of biopsies evaluated, one colon cancer patient with stable disease had γH2AX and pNBS1 above the previously defined assay baseline of 4% nuclear area positive (NAP)1 in biopsies collected 18–22 hours after the end of irinotecan infusion and increased induction of γH2AX and pNBS1 18–22 hours after the end of irinotecan and berzosertib combination treatment (Figure 3C,D) indicative of target engagement.

### Tumor genomic analysis

**FIGURE 4:**
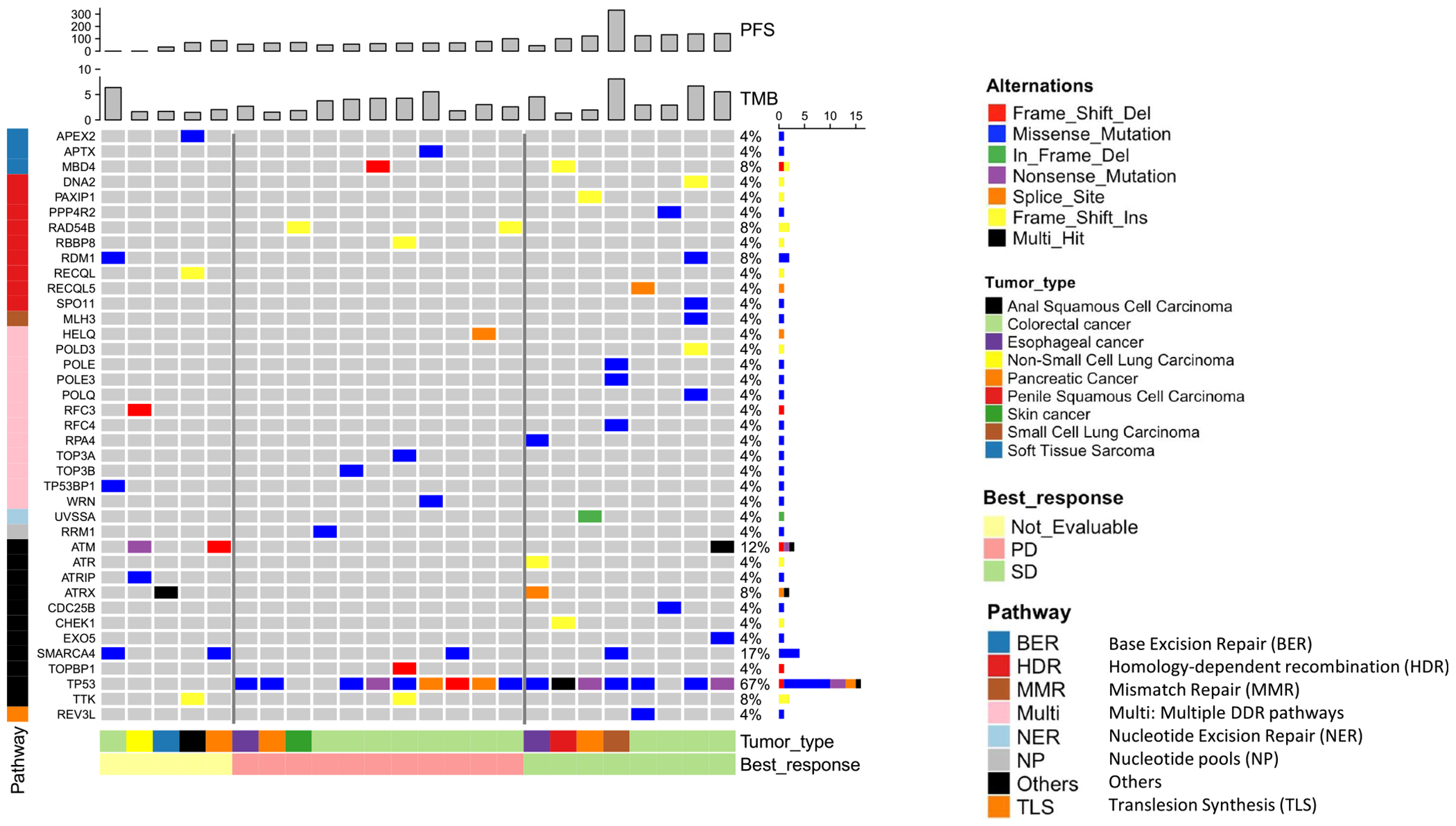
Presence of DNA damage response gene variants by whole exome sequencing of archival tumor tissue, sorted by best response.

Of 31 archival tumor specimens received for WES, 28 passed quality control and 27 of these had matched peripheral blood controls. WES was performed on 27 matched specimens, including 12 with PD, 10 with SD, and five who were not evaluable for response. The tumor specimens from two patients with PR were not included in the analysis because they were not available. The SD group had a higher prevalence of DDR gene mutations and OncoKB-annotated cancer gene mutations compared with the PD group (Figure 4; Supporting Information S1: Figure S6). The median tumor mutational burden (TMB) was 3.71 mut/Mb for the SD group, 2.95 mut/Mb for the PD group, and 1.69 mut/Mb for the not evaluable group. ATM mutations were present in one patient with SD and two patients who were not evaluable for response (Figure 4). Aneuploidy score (number of chromosome arms with aneuploidy) was higher in the SD group compared with the PD group, although this was not significant (Supporting Information S1: Figure S7). Copy number gain of chromosome 13q and *BRCA2* was more prevalent in SD (40%) compared with PD (25%) (Supporting Information S1: Figure S8). There was no difference in copy number gain or loss of DDR genes between SD and PD groups overall (Supporting Information S1: Figure S9).

## DISCUSSION

This study explored the safety, antitumor activity, PK, and pharmacodynamics of the novel combination of irinotecan and berzosertib in advanced solid tumors. The MTD and RP2D were determined to be berzosertib 270 mg/m^2^ and irinotecan 180 mg/m^2^ every 2 weeks. The most common adverse events (myelosuppression, nausea, vomiting, and diarrhea) were consistent with the known toxicity profile of irinotecan,^18^ although rates of grade 3 and 4 toxicities were higher with the current combination compared with other studies evaluating biweekly irinotecan in combination with cetuximab.^19,20^ Two among 45 patients experienced DLTs in the form of febrile neutropenia at the MTD. Notably, berzosertib was dosed on the same day as the chemotherapeutic agent in our study rather than the day after as in other studies, based on preclinical data suggesting that the optimal timing of berzosertib is up to 4 hours after irinotecan administration.^21–23^ This schedule did not lead to intolerable toxicity or compromise the ability to escalate to the estimated minimum therapeutic human dose (100 mg/m^2^) and it is more convenient than sequential days of dosing.

Observed values for berzosertib clearance, volume of distribution, and half-life were comparable to those reported in a recent population pharmacokinetic analysis,^24^ although the clinically insignificant increase in distribution volume and half-life with dose was not previously reported. Although saturated plasma protein binding could theoretically explain this, concentrations required were not achieved systemically and may have only briefly been achieved locally during infusion. The reported human equivalent p-Chk1 IC_50_ of approximately 110 ng/mL was exceeded at all DLs, and for more than 24 h at the MTD of 270 mg/m^2^. The pharmacokinetics of irinotecan and metabolites were in line with a previous report except our NPC AUC was approximately half the reported value and SN38G AUC was approximately 1.46-fold the reported value.^25^ The coad-ministration of berzosertib had no impact on exposure of irinotecan or active metabolite SN38. Even with a statistically significant 21% decrease in NPC/irinotecan metabolic ratio, berzosertib was not found to result in clinically significant changes in irinotecan exposure or metabolism. In our relatively large data set, we did not detect a relationship between UGT1A1 genotype and SN38 or SN38G/SN38 ratio, and the statistically significant finding of day −14 SN38G AUC with UGT1A1 genotype was driven by an increase in SN38G from *1/*1 to *1/*28, which is counter to the biological mechanism of *28 alleles resulting in reduced glucuronidation. Our inability to detect the expected increase in SN38 with *28 alleles and consequent increase in toxicity associated with SN38 exposure may be explained by our relatively low dose of 180 mg/m^2^ irinotecan. Several reports suggest that the presence of the UGT1A1*28 allele is not a major risk factor for toxicity at doses at or below 180 mg/m^2^.^26,27^ Interestingly, berzosertib AUC was significantly associated with highest grade any toxicity and hematologic-specific toxicity.

Despite heavy pretreatment with exposure to a median of four prior therapies, two pancreatic cancer patients treated with berzosertib and irinotecan achieved a PR, and 22 patients had SD. Both PRs lasted over 12 months and occurred in patients with metastatic pancreatic cancer (PDAC) and ATM gene alterations on local tumor NGS (ATM E11828/ATM K1109* and ATM R3008H/germline ATM R1882*). ATM is one of the most commonly mutated genes in the homologous recombination deficiency (HRD) pathway and has an estimated 5% prevalence in advanced PDAC.^28–30^ Interestingly, pathogenic ATM mutations have been associated with a favorable prognosis in PDAC.^31,32^ In addition to the partial responses, a CRC patient with an ATM mutation identified on WES experienced SD lasting 4.7 months and had induction of DDR markers with addition of berzosertib to irinotecan on pharmacodynamic analysis. The presence of ATM alterations in both partial responders and in a patient with stable disease with evidence of treatment-associated DNA damage supports the hypothesis that cancer cells with deficient ATM-p53 signaling are more responsive to ATR inhibition combined with chemotherapy.^3,4^

Mutations in TP53 and other DDR genes, including BRCA1/2, ATM, ARID1A, and CHEK1/2, have been identified in responders across phase 1 trials of berzosertib in combination with other chemotherapeutic agents (cisplatin, carboplatin, gemcitabine ± cisplatin, and topotecan) in advanced solid tumors.^7,21–23^ These results suggest that existing vulnerabilities in the DDR may promote a reliance on ATR for cell survival, leading to synthetic lethality when treated with ATR inhibitors and chemotherapy. Notably, in our trial, patients with SD harbored more DDR mutations in tumor tissue compared with patients with PD. However, this does not necessarily imply that patients with DDR mutations derive greater benefit from berzosertib and irinotecan, as SD patients also had a higher TMB and aneuploidy score, suggesting greater genomic instability in general. Copy number gain of *BRCA2* was noted in a higher proportion of SD patients than PD patients. The significance of this is unclear. Prior studies have linked *BRCA2* copy number loss, but not copy number gain, with chromosomal instability and early biochemical recurrence in prostate cancer, as well as increased risk of developing ovarian cancer.^33–35^

In a phase 2 trial, the combination of berzosertib and gemcitabine improved PFS in recurrent platinum-resistant high-grade serous ovarian carcinoma (HGSOC) compared with gemcitabine alone.^36^ HGSOC is characterized by near universal G1/S checkpoint loss via TP53 mutation.^37^ Notably, a subset of patients who had negative ATM expression in tumor cells had an OS benefit that was not found in ATM-positive patients.^38^ SCLC has similarly been found to have a high rate of TP53 and RB1 inactivation, but a phase 2 trial failed to show a PFS benefit of berzosertib and topotecan over topotecan alone.^39,40^ In a phase 2 clinical trial comparing gemcitabine and cisplatin plus berzosertib with gemcitabine and cisplatin alone, the addition of berzosertib resulted in inferior survival compared with chemotherapy alone in patients with metastatic urothelial cancer.^41^ Notably, there were higher rates of grade 3 and 4 thrombocytopenia and neutropenia and more dose reductions in the berzosertib containing arm resulting in a statistically significant lower exposure to cisplatin, which may explain the inferior outcomes in the berzosertib containing arm. Elimusertib, a more potent, selective ATR inhibitor, has been associated with prohibitive myelotoxicity when combined with FOLFIRI in patients with advanced gastrointestinal malignancies.^42^ In vitro studies have demonstrated that elimusertib is associated with longer persistence of ATR pathway inhibition compared with the ATR inhibitor ceralasertib.^42^ These studies suggest the ability to combine ATR inhibitors with chemotherapy is dependent both on the degree of myelotoxicity of the chemotherapy backbone and the depth and duration of pathway inhibition with the ATR inhibitor.

We explored pharmacodynamic biomarkers of DNA DSBs in both peripheral blood and tumor tissue in response to treatment. In PBMCs, γH2AX increased 10-fold on average after administration of irinotecan alone, with no noticeable difference with addition of berzosertib. Results must be interpreted with caution as PBMC count unexpectedly dropped by 70% in the hours after treatment, returning back to baseline after 24 h. Likely this represents the well-described redistribution of lymphocytes from the blood to the bone marrow after steroid administration, which was a component of the premedication regimen for the study treatment.^43^

In tumor tissue collected 18–22 hours after treatment, berzosertib and irinotecan led to an increase of γH2AX and pNBS1 levels above irinotecan alone in a CRC patient treated at DL4 who achieved a best response of SD. Although this signal was only identified in one of five evaluable paired biopsies, it supports the hypothesis that ATR inhibition with berzosertib can exacerbate irinotecan-induced DNA damage to tumor cells. Preclinical data suggests sustained accumulation of DNA damage markers γH2AX and pS824-KAP1 in cell lines exposed to DNA-damaging agents and berzosertib, and pS343-NBS1 is another DNA damage biomarker that has been validated in xenograft mice and tumor specimens.^14^ γH2AX induction in hair follicles has been reported in patients treated with berzosertib and topotecan, but to our knowledge, this is the first clinical study demonstrating the accumulation of γH2AX and pS343-NBS1 in tumor tissue with addition of an ATR inhibitor to a chemotherapeutic agent.^22^

In conclusion, the combination of irinotecan and berzosertib was associated with no unexpected toxicities in patients with advanced solid tumors and resulted in durable partial responses in two patients with pancreatic cancer harboring ATM mutations. Although no further studies with berzosertib are planned in favor of orally bioavailable ATR inhibitors, such as tuvusertib,^44^ our clinical trial validates ongoing efforts evaluating ATR inhibitors across tumor types, in combination with chemotherapy (NCT05691491), PARP inhibitors (NCT05269316, NCT06433219), immunotherapy (NCT06518564 and NCT05947500), and novel agents (NCT05950464 and NCT05687136). Additionally, several trials (NCT04065269 and NCT03682289) have included prespecified cohorts with ATM or ARID1A abnormalities. Our study supports these investigations into molecular subpopulations that may benefit most from ATR inhibitors.

## Supplementary Material

s1

Additional supporting information can be found online in the Supporting Information section at the end of this article.

## Figures and Tables

**TABLE 1:** Patient characteristics.

Characteristic	No. (%)
No. of patients	63 (100)
Sex	
M	31 (49)
F	32 (51)
Age (years), median (range)	59 (26–77)
Race	
Asian	5 (8)
Black/African American	3 (5)
Not reported	4 (6)
White	51 (81)
ECOG	
0	19 (30)
1	44 (70)
Tumor type	
Colorectal	31 (49)
Pancreatic	13 (21)
Small-cell lung	3 (5)
Non–small cell lung	3 (5)
Esophageal	2 (3)
Biliary	2 (3)
Other	9 (14)
Prior therapies (median, range)	4 (2–7)
UGT1A1	
*1/*1 (negative for both)	34 (54)
*1/*28 (positive)	21 (33)
*28/*28 (positive)	6 (10)
Unknown	2 (3)

Abbreviations: ECOG, Eastern Cooperative Oncology Group; F, female; M, male.

**TABLE 2:** Most common (≥10%) treatment-related adverse event (at least possibly related, maximum grade, all cycles).

Toxicity (CTCAE v5.0 term)	All (*N* = 63)		DL1 (*N* = 1)		DL2 (*N* = 9)		DL3 (*N* = 8)		DL4 (*N* = 45)	
Adverse event	All	**≥Grade 3**	All	≥Grade 3	All	≥Grade 3	All	≥Grade 3	All	≥Grade 3
HEME, No. (%)										
Anemia	36 (57)	16 (25)	1 (100)		5 (56)	2 (22)	3 (38)	2 (25)	27 (60)	12 (27)
WBC decrease	33 (52)	14 (22)	1 (100)		3 (33)		2 (25)	1 (13)	27 (60)	13 (29)
Neutrophil decrease	31 (49)	18 (29)			2 (22)	1 (11)	2 (25)	2 (25)	27 (60)	15 (33)
Lymphocyte decrease	34 (54)	19 (30)	1 (100)	1 (100)	4 (44)	3 (33)	3 (38)	2 (25)	26 (58)	13 (29)
Platelet decrease	16 (25)	2 (3)			1 (11)		2 (25)		13 (29)	2 (4)
Non-HEME, No. (%)										
Abdominal pain	10 (16)				3 (33)		2 (25)		5 (11)	
Alopecia	12 (19)				2 (22)		2 (25)		8 (18)	
ALK increase	13 (21)				4 (44)				9 (20)	
ALT increase	7 (11)						1 (13)		6 (13)	
Anorexia	9 (14)	1 (2)	1 (100)		2 (22)		1 (13)		5 (11)	1 (2)
AST increase	9 (14)						2 (25)		7 (16)	
Diarrhea	33 (52)	11 (16)			4 (44)	1 (11)	4 (50)	2 (25)	25 (56)	8 (18)
Dizziness	6 (10)				1 (11)		1 (13)		4 (9)	
Fatigue	20 (32)	4 (6)	1 (100)	1 (100)	1 (11)		3 (38)		15 (33)	3 (7)
Hypokalemia	10 (16)	4 (6)			4 (44)	2 (22)	3 (38)	1 (13)	3 (7)	1 (2)
Hypomagnesemia	6(10)				2 (22)				4 (9)	
Hyponatremia	12 (19)				3 (33)		2 (25)		7 (16)	
Hypophosphatemia	6(10)	2 (3)			3 (33)	2 (22)			3 (7)	
Muscle weakness	6 (10)				1 (11)		1 (13)		4 (9)	
Nausea	26 (41)	2 (3)	1 (100)		5 (56)		4 (50)		16 (36)	2 (4)
Vomiting	23 (37)	3 (5)	1 (100)		6 (67)		1 (13)		15 (33)	3 (7)
Weight loss	9 (14)		1 (100)		1 (11)		2 (25)		5 (11)	

Abbreviations: ALK, anaplastic lymphoma kinase; ALT, alanine aminotransferase; AST, aspartate aminotransferase; CTCAE, Common Terminology Criteria for Adverse Events; DL3, dose level 3; DL4, dose level 4; WBC, white blood cell.

## Data Availability

The data generated in this study are publicly available in ClinicalTrials.gov (NCT02595931).
