# Supplementary material for: Phase 1 clinical trial of the ataxia telangiectasia and Rad3-related inhibitor berzosertib with irinotecan in patients with advanced solid tumors (ETCTN 9938)
Source: Cancer. Author manuscript; Available in PMC 2026 Mar 2. (PMC12952245; doi:10.1002/cncr.70157)
Supplement: s1 [file NIHMS2144431-supplement-s1.docx]

# SUPPLEMENTARY

## FIGURES


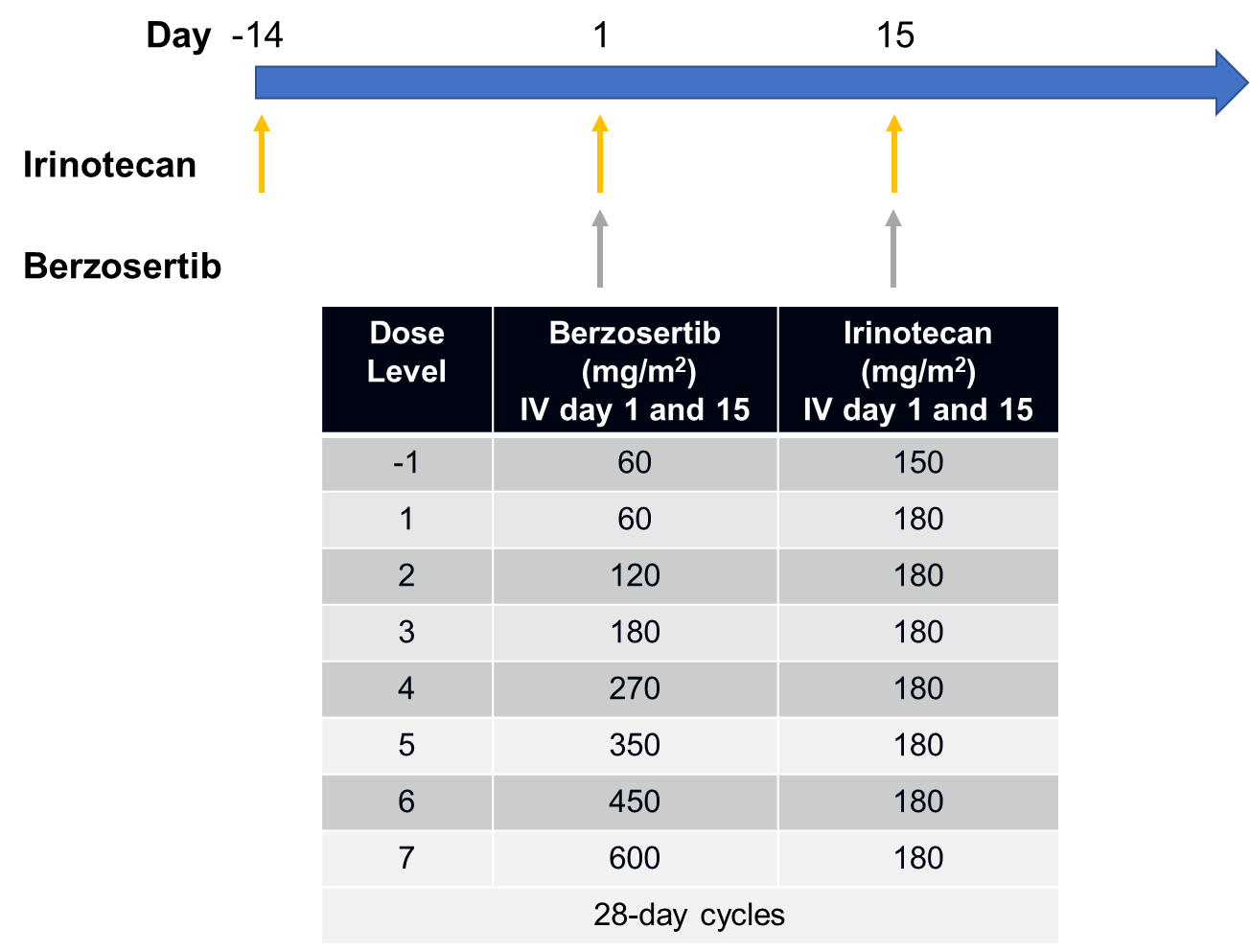


Figure S1. Trial schema and dose escalation schedule. IV, intravenously.


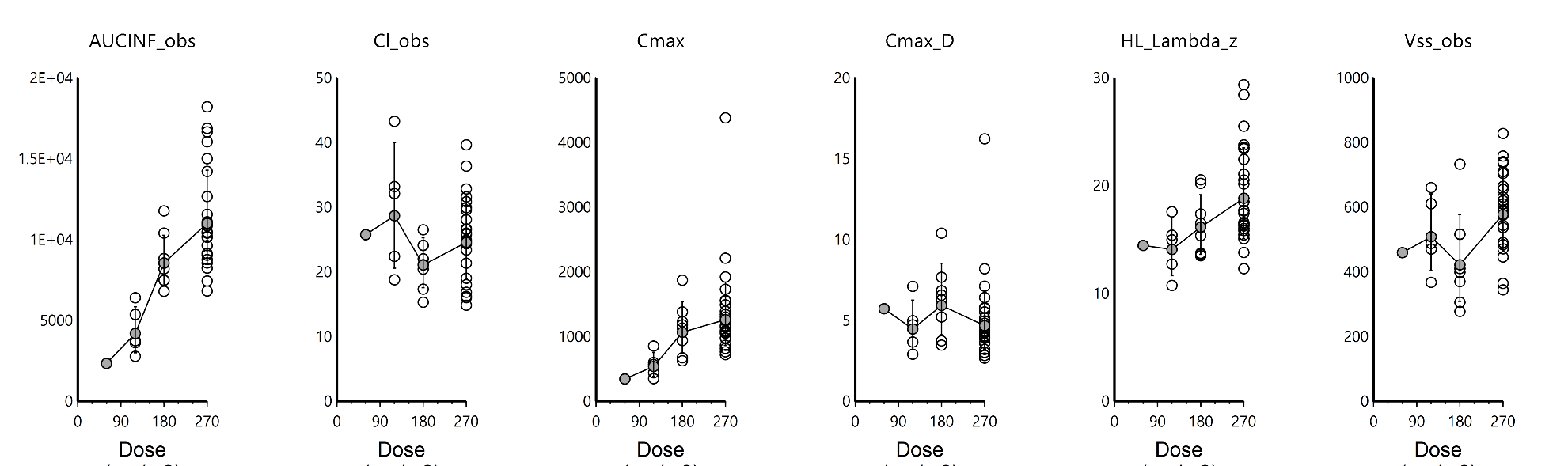


Figure S2. Berzosertib PK parameters (geometric mean, SD) as a function of dose (mg/m^2^). AUC_0-inf_ (µg/L•h), clearance (L/h/m^2^, *P*=0.693), C_max_ (µg/L), dose-normalized C_max_ (µg/L / (mg/m^2^), *P*=0.721), half-life (h, *P*=0.005), and volume of distribution at steady-state (L/m^2^, *P*=0.025). *P* values by general linear model with dose as covariate.

Figure S3. Range of berzosertib human plasma protein binding from 300 – 100,000 µg/L. Lines represent geometric mean

Figure S4. Association of day -14 AUC for SN38 (p=0.059), SN38G (p=0.026), and the ratio of SN38G/SN38 (p=0.603) with UGT1A1 genotype (1=*1/*1; 2=*1/*28; 3=*28/*28); individual data with geometric mean (±geoSD).

Figure S5. Association of day 15 berzosertib AUC with at least possibly attributed highest grade of any toxicity (p=0.035), highest grade heme toxicity (p=0.032), and highest grade non-heme toxicity (p=0.561) by Jonckheer-Terpstra.


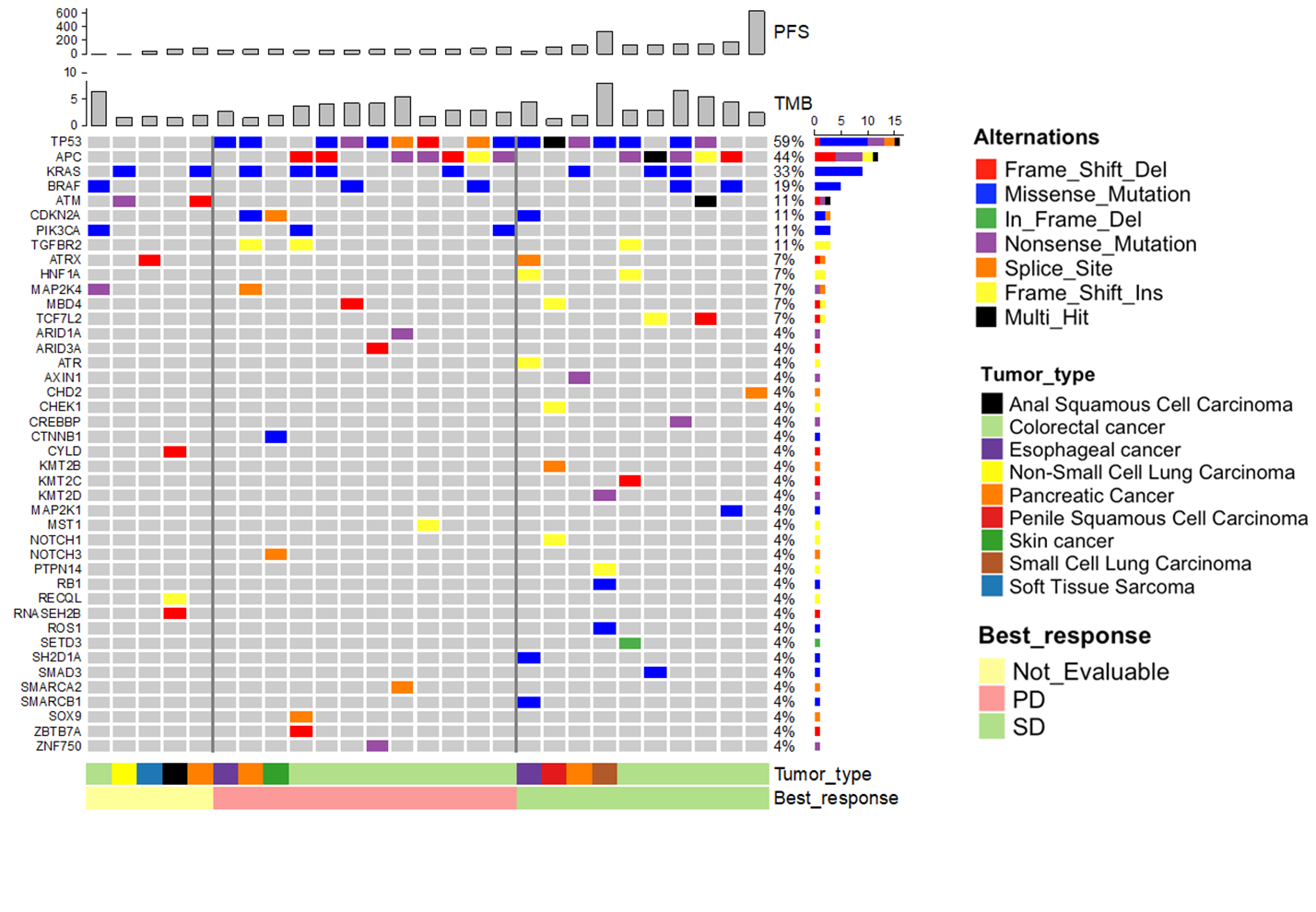


Figure S6. Presence of OncoKB annotated cancer gene variants in archival tumor tissue, sorted by best response.


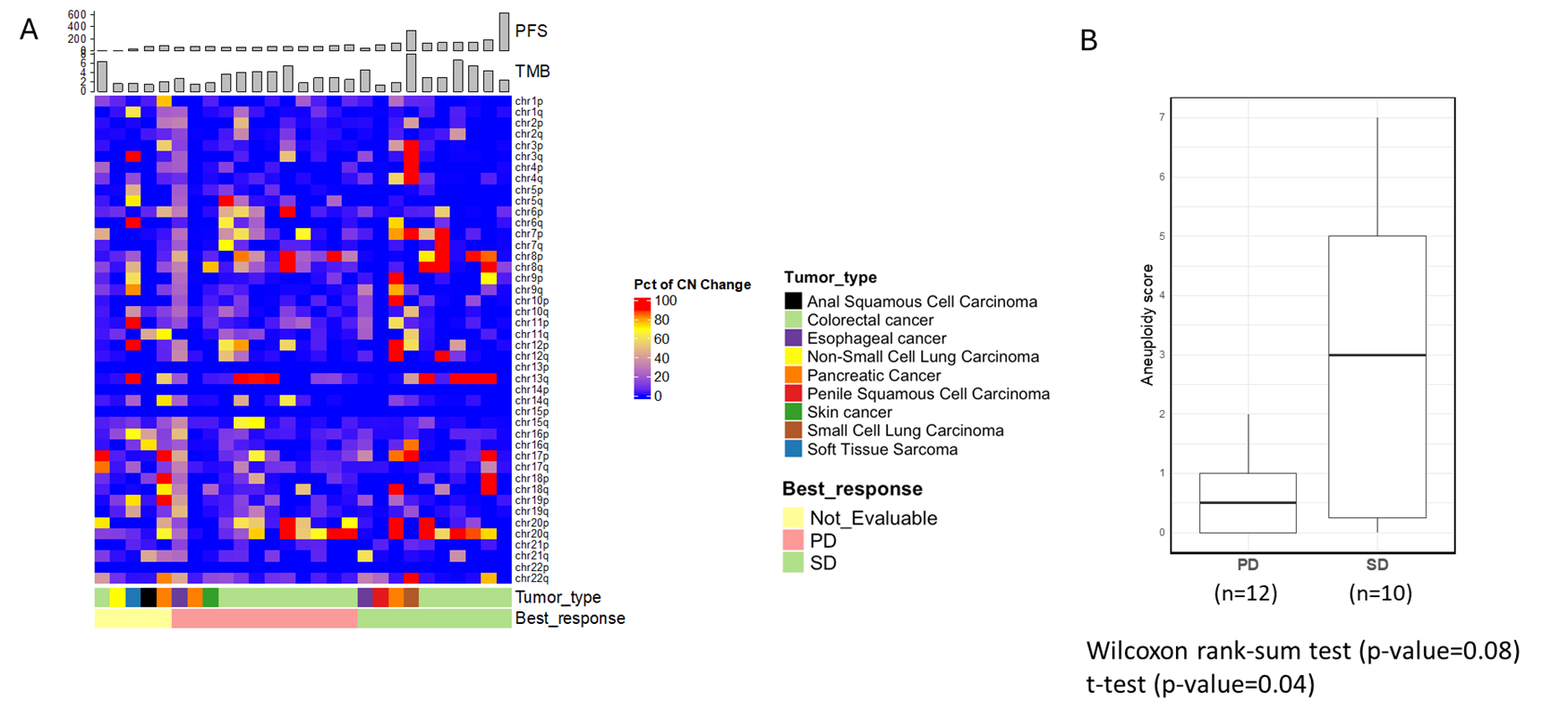


Figure S7. Aneuploidy score analysis from whole-exome sequencing of archival tumor tissue. (A) Percent copy number change in chromosome arms by best response. (B) Number of chromosome arms with aneuploidy (≥90% of arm demonstrating copy number gain or loss) was greater in the SD group than the PD group.


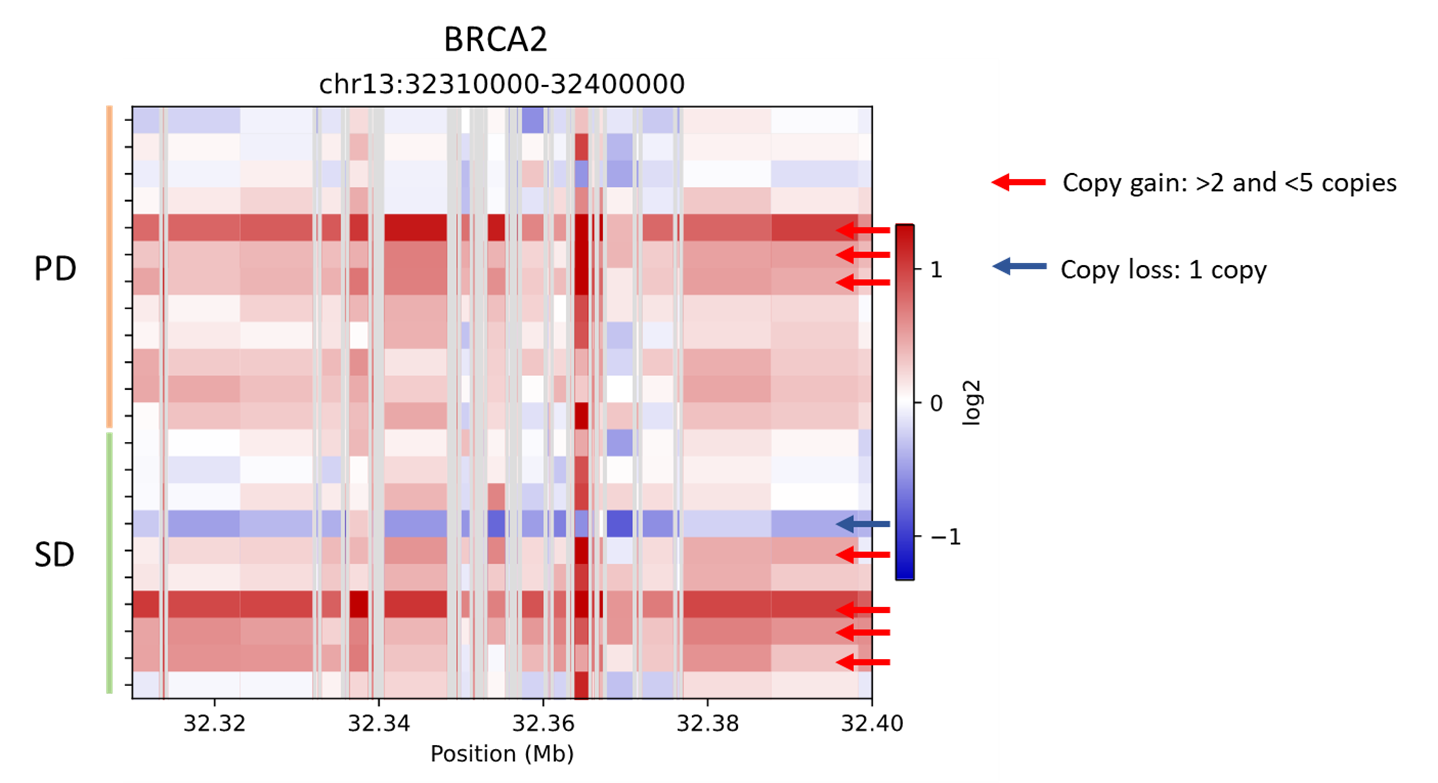


Figure S8. BRCA2 copy number gain found in 40% (4/10) of SD patients and 25% (3/12) of PD patients.


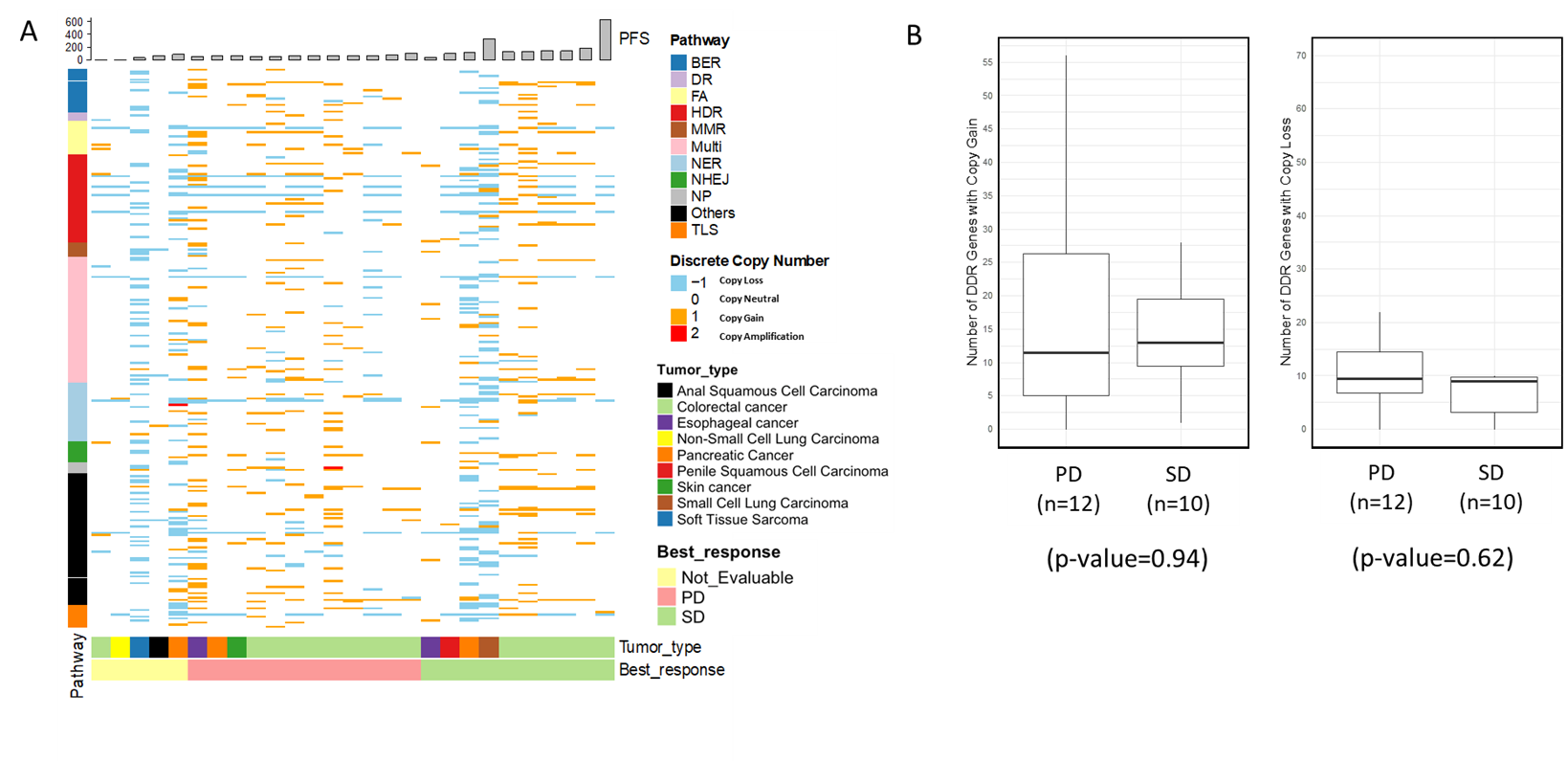


Figure S9. (A) Copy number profile for DDR genes, sorted by best response. (B) No difference in copy number gain or loss of DDR genes was found between PD and SD groups.

## TABLES

Table S1. Berzosertib geometric mean (SD) plasma pharmacokinetic parameters for day 15 of cycle 1.

| Dose  (mg/m^2^) | C_max_  (µg/L) | C_max_/Dose  (µg/L / mg/m^2^) | T_max_  (h) | t½  (h) | AUC_0-inf_^a^  (mg/L•h) | Cl  (L/h/m^2^) | V_ss_^b^  (L/m^2^) |
| --- | --- | --- | --- | --- | --- | --- | --- |
| 60 (N=1) | 343 (-) | 5.72 (-) | 0.45 (-) | 14.4 (-) | 2.33 (-) | 25.7 (-) | 459 (-) |
| 120 (N=5) | 535 (1.40) | 4.46 (1.40) | 0.54 (2.2) | 14.1 (1.2) | 4.19 (1.40) | 28.7 (1.40) | 508 (1.26) |
| 180 (N=8) | 1065 (1.44) | 5.92 (1.44) | 0.66 (2.0) | 16.1 (1.2) | 8.55 (1.20) | 21.1 (1.20) | 422 (1.37) |
| 270 (N=25) | 1260 (1.46) | 4.67 (1.46) | 0.61 (2.5) | 18.8 (1.3) | 11.0 (1.30) | 24.6 (1.30) | 578 (1.24) |
| Total (N=39) | - | 4.90 (1.45) | 0.61 (2.3) | 17.5 (1.3) | - | 24.3 (1.30) | 530 (1.30) |

SD, geometric standard deviation.

^a^AUC_0-inf_ extrapolated beyond the last time point sampled was less than 17.8% (geometric mean 5.8%).

^b^V_z_, overall 612 (1.31) L/m^2^

Table S2. Irinotecan and metabolites geometric mean (SD) plasma pharmacokinetic parameters for days -14 and 15 of cycle 1.

|  |  |  | Irinotecan |  |  |  |  | SN38 |  |  |
| --- | --- | --- | --- | --- | --- | --- | --- | --- | --- | --- |
|  | D-14 | D-14 | D-14 | D-14 | D-14 | D15/D-14 | D-14 | D-14 | D-14 | D15/D-14 |
| Dose  (mg/m^2^) | C_max_  (µg/L) | t½  (h) | AUC_0-inf_^a^  (mg/L•h) | Cl  (L/h/m^2^) | V_ss_^b^  (L/m^2^) | AUC^c^  Ratio (N=39) | C_max_  (µg/L) | t½  (h) | AUC_0-inf_^a^  (mg/L•h) | AUC^c^  Ratio |
| 180 (N=55) | 2181 (1.28) | 12.9 (1.2) | 14.0 (1.52) | 12.9 (1.52) | 126 (1.36) | 0.999 (1.32) | 25.0 (1.78) | 19.9 (1.3) | 0.38 (1.8) | 1.07 (1.7) |
|  |  |  |  |  | Average BE^c^ | 100.0% |  |  |  | 106.6% |
|  |  |  |  |  | L-90%CI | 93.0% |  |  |  | 93.6% |
|  |  |  |  |  | U-90%CI | 107.6% |  |  |  | 121.5% |

SD, geometric standard deviation. BE, Bioequivalence assessment. L-90%CI, lower 90% confidence interval. U-90%CI, upper 90% confidence interval

^a^AUC_0-inf_ extrapolated beyond the last time point sampled was geometric mean 1.0% (range 0.3-4.4%) for irinotecan and 7.9% (range 2.0-32%) for SN38.

^b^V_z_, overall 240 (1.55) L/m^2^

^c^to account for dose decreases to 150 mg/m^2^ on day 14 in 4 patients, dose-normalized AUC values were used,

Table S3. Irinotecan metabolites geometric mean (SD) plasma pharmacokinetic parameters for day -14 of cycle 1.

|  |  | APC |  |  | NPC |  |  | SN38G |  |
| --- | --- | --- | --- | --- | --- | --- | --- | --- | --- |
| Dose  (mg/m^2^) | C_max_  (µg/L) | t½  (h) | AUC_0-inf_^a^  (mg/L•h) | C_max_  (µg/L) | t½  (h) | AUC_0-inf_^a^  (mg/L•h) | C_max_  (µg/L) | t½  (h) | AUC_0-inf_^a^  (mg/L•h) |
| 180  (N=55) | 201  (1.81) | 12.1  (1.3) | 2.74  (1.92) | 11.8  (2.00) | 12.5  (1.6) | 0.124  (2.22) | 167  (1.63) | 18.8  (1.29) | 2.89  (1.84) |

SD, geometric standard deviation.

^a^AUC_0-inf_ extrapolated beyond the last time point sampled was geometric mean 1.5% (range 0.2-14.3%) for APC, 5.3% (range 0.2-48.6%) for NPC, and 6.9% (range 1.6-22.9%) for SN38G.

Table S4. Irinotecan metabolites geometric mean (SD) plasma pharmacokinetic parameters for days -14 and 15 of cycle 1.

|  | SN38G/  SN38 | | NPC/  IRI | | APC/  IRI | | SN38/  IRI | |
| --- | --- | --- | --- | --- | --- | --- | --- | --- |
|  | D-14 | D15 | D-14 | D15 | D-14 | D15 | D-14 | D15 |
| Dose  (mg/m^2^) | AUC  Ratio | AUC  Ratio | AUC  Ratio | AUC  Ratio | AUC  Ratio | AUC  Ratio | AUC  Ratio | AUC  Ratio |
| 180  (N=55) | 7.72 (1.74) |  | 0.0088 (1.96) |  | 0.196 (1.80) |  | 0.0268 (1.56) |  |
| 180/150  (N=41) |  | 8.55 (1.78) |  | 0.0074 (2.26) |  | 0.221 (1.96) |  | 0.0282 (1.70) |
| D15/D-14 (N=39) | - | 1.05 (1.49) | - | 0.79 (1.77) | - | 1.09 (1.50) | - | 1.07 (1.48) |
| P-value^a^ |  | 0.49 |  | 0.005 |  | 0.46 |  | 0.16 |

SD, geometric standard deviation.

^a^ metabolic ratios were compared between days -14 and 15 by the paired Wilcoxon exact sign test (2-tailed).

Table S5. UGT1A1 status and pharmacokinetics.

|  | Day | SN38 AUC | SN38G AUC | SN38G/SN38 | berzo C_max_/Dose | berzo CL |
| --- | --- | --- | --- | --- | --- | --- |
| UGT1A1  *1/*1 - *1/*28 - *28/*28 | -14 | 0.059 | 0.026 | 0.603 | - | - |
|  | 15 | 0.622 | 0.138 | 0.458 | 0.245 | 0.75 |

By exact Jonckheere-Terpstra test with UGT1A1 genotype as ordinal variable.

Table S6. Irinotecan and berzosertib exposure-toxicity relationships.

| Ordinal | Day | IRI AUC | SN38 AUC | SN38G AUC | SN38G/SN38 | berzo C_max_ | berzo AUC |
| --- | --- | --- | --- | --- | --- | --- | --- |
| highest grade tox  0-4 | Lead-in  (D-14 – 0)  D-14 PK | 0.125 | 0.080 | 0.163 | 0.726 | - | - |
| highest grade heme tox  0-4 |  | 0.104 | 0.144 | 0.169 | 0.876 | - | - |
| highest grade non-heme tox  0-4 |  | 0.715 | 0.461 | 0.800 | 0.812 | - | - |
| highest grade tox  0-4 | Cycle 1  (D1-28)  D15 PK | 0.052 | 0.387 | 0.148 | 0.094 | 0.630 | 0.035 |
| highest grade heme tox  0-4 |  | 0.153 | 0.358 | 0.314 | 0.194 | 0.425 | 0.032 |
| highest grade non-heme tox  0-4 |  | 0.058 | 0.605 | 0.228 | 0.718 | 0.960 | 0.561 |

By exact Jonckheere-Terpstra test with highest grade toxicity (at least possibly related) as ordinal variable.

Table S7. UGT1A1 status and toxicity relationships.

|  | Day | highest grade tox  0-4 | highest grade heme tox  0-4 | highest grade non-heme tox  0-4 |
| --- | --- | --- | --- | --- |
| UGT1A1  *1/*1 - *1/*28 - *28/*28 | Lead-in  (D-14 – 0) | 0.730 | 0.699 | 0.751 |
|  | Cycle 1  (D1-28) | 0.0167* | 0.2322 | 0.0224* |

By Fisher’s Exact test.

*increasing grade of highest toxicity with *28 alleles relative to *1/*1.
